# Correction: Imaging cytoplasmic lipid droplets in vivo with fluorescent perilipin 2 and perilipin 3 knock-in zebrafish

**DOI:** 10.7554/eLife.91723

**Published:** 2023-08-09

**Authors:** Meredith H Wilson, Stephen C Ekker, Steven A Farber

**Keywords:** Zebrafish

Wilson MH, Ekker SC, Farber SA. 2021. Imaging cytoplasmic lipid droplets in vivo with fluorescent perilipin 2 and perilipin 3 knock-in zebrafish. *eLife* **10**:e66393. doi: 10.7554/eLife.66393.

Published 13 August 2021

There was an error in the Materials and methods of this article. We inadvertently omitted a citation to the following publication, which served as a guide for our genome editing methodology. This citation has now been included in the first sentence of the section “Genome editing to create PLIN fusion lines”.

Shin, J., Chen, J. & Solnica-Krezel, L. Efficient homologous recombination- mediated genome engineering in zebrafish using TALE nucleases. Development 141, 3807–3818 (2014). DOI: https://doi.org/10.1242/dev.108019; PMID: 25249466

Corrected text: Fus(EGFP-plin2) and Fus(plin3-RFP) lines were created with TALEN-mediated genome editing, using (Shin et al., 2014) as a guide.

Original text: *Fus(EGFP-plin2*) and *Fus(plin3-RFP*) lines were created with TALEN-mediated genome editing.

Additionally, the knock-in reporter lines are now available from the Zebrafish International Resource Center (ZIRC): plin2^c847Tg^ (ZIRC Catalog ID: ZL14605) and plin3^875Tg^ (ZIRC Catalog ID: ZL14606).

The article has been corrected accordingly.

