# Supplementary material for: Imaging cytoplasmic lipid droplets in vivo with fluorescent perilipin 2 and perilipin 3 knock-in zebrafish
Source: eLife. 2021 Aug 13;10:e66393. doi: 10.7554/eLife.66393 (PMC8460263; doi:10.7554/eLife.66393)
Supplement: Supplementary file 1. [file elife-66393-supp1.docx]

**Supplementary File 1: Primers**

| **Gene/Figure #** | **Purpose** | **Sequence** |
| --- | --- | --- |
| *plin2* Fig 1A,B | *In situ* probe | F: CGT GCA AAG GAC TGG ATA AG |
| *plin2* Fig 1A,B | *In situ* probe | R: AGA CCC CTG AGA CTG GAC AC |
| *plin3* Fig 1A,B | *In situ* probe | F: AGA CCG ACT GGA ACC TCA GA |
| *plin3* Fig 1A,B | *In situ* probe | R: CTG GCG TGT CTG CAG TAA GA |
| *plin2* Fig 1 – S1 F1 | TALEN target site verification | F: TGC ACC TTA AAC TCA AAC CGT G |
| *plin2* Fig 1 – S1 R1 | TALEN target site verification | R: AGG ATT AAA GTG GCA AAC CTG G |
| *plin2* Fig 1 – S1 | qRT-PCR | F: TTC ACT AAT GGG CTG GAA GA |
| *plin2* Fig 1 – S1 | qRT-PCR | R: CAC CAC ACA TGT GCT CTG AA |
| *rps18* Fig 1- S1 | qRT-PCR | F: TGC AGA ACC CTC GCC AGT ACA AAA TCC CAG |
| *rps18* Fig 1 – S1 | qRT-PCR | R: CCA GAA GTG ACG GAG ACC ACG GTG AGC CCT |
| *plin2* | Amplification of left homology arm for donor plasmid | F: GGG GAC AAC TTT GTA TAG AAA AGT TGA AGC CCT GAT ACA ACA TAT TCG C |
| *plin2* | Amplification of left homology arm for donor plasmid | R: GGG GAC TGC TTT TTT GTA CAA ACT TGA GTT AGC AGA AAA TCT GCA AAA G |
| *plin2* | Amplification of right homology arm for donor plasmid | F: GGG GAC AGC TTT CTT GTA CAA AGT GGA AAT GGG TTC TAT GGA GGA TGT |
| *plin2* | Amplification of right homology arm for donor plasmid | R: GGG GAC AAC TTT GTA TAA TAA AGT TGA GTG ATT GGA TGT GTT TTG GAT TG |
| *EGFP* | pME EGFP for donor plasmid | F: GGG GAC AAG TTT GTA CAA AAA AGC AGG CTG CTA ACA TGG TGA GCA AGG GCG AGG AGC TGT |
| *EGFP* | pME EGFP for donor plasmid | GGG GAC CAC TTT GTA CAA GAA AGC TGG GTG TCC ACC GCC CTT GTA CAG CTC GTC CAT GCC GAG A |
| *plin3* | Amplification of left homology arm for donor plasmid | F: GGG GAC AAC TTT GTA TAG AAA AGT TGA ACC AGC AGA TTG GCC AGG TAG |
| *plin3* | Amplification of left homology arm for donor plasmid | R: GGG GAC TGC TTT TTT GTA CAA ACT TGA TTC ACC TTT CTC TGT TAT CTG AGG |
| *plin3* | Amplification of right homology arm for donor plasmid | F: GGG GAC AGC TTT CTT GTA CAA AGT GGA AAA TTG CCA CAC AAC CTA AAT AAA TCT G |
| *plin3* | Amplification of right homology arm for donor plasmid | R: GGG GAC AAC TTT GTA TAA TAA AGT TGA ACT TCT TCA TAG AAT CCT GTG TCC A |
| *tagRFP-t* | pME tagRFP-t for donor plasmid | F: GGG GAC AAG TTT GTA CAA AAA AGC AGG CTT GAT GGT GAG CAA AGG AGA GGA AC |
| *tagRFP-t* | pME tagRFP-t for donor plasmid | R: GGG GAC CAC TTT GTA CAA GAA AGC TGG GTT TAC TTG TAC AGC TCA TCC ATT CC |
| *plin2* Fig 1C F1 | Integration confirmation PCR | F: TGC TGA AGA AGA GTG ATC TCA TCC |
| *plin2* Fig 1C R1 | Integration confirmation PCR | R: GTG CGC TCC TGG ACG TAG CCT TCG |
| *plin2* Fig 1C F2 | Integration confirmation PCR | F: CAA GGA GGA CGG CAA CAT CCT GGG |
| *plin2* Fig 1C R2 | Integration confirmation PCR | R: AAA TGT TTG CAC ATC AGA CTA CAG |
| *plin2* Fig 1C F3 | Integration confirmation PCR | F: TGG TCT CAG CGT GAA ATC CC |
| *plin2* Fig 1C R3 | Integration confirmation PCR | R: TCC TTG CTT TGT CAA CCT ACC A |
| *plin3*  Fig 1C F4 | Integration confirmation PCR | F: AGG AAC AGC TTC TCA ATG CTC G |
| *plin3* Fig 1C R4 | Integration confirmation PCR | R: GCA TCA CAG GTC CAT TGC TA |
| *plin3* Fig 1C F5 | Integration confirmation PCR | F: CTT CCC TAG CAA TGG ACC TG |
| *plin3* Fig 1C R5 | Integration confirmation PCR | R: AAC TAT TCG GTG GCG CAG AA |
|  |  |  |
| *plin3* Fig 1C F6 | TALEN target site verification &  Integration confirmation PCR | F: CGG CAG TCT CTT GAT GGA GT |
| *plin3* Fig 1C R6 | TALEN target site verification &  Integration confirmation PCR | R: ACA ACA ACT ATA AAG TAT GGC TTG C |
| *EGFP* | Genotyping *Fus(EGFP-plin2)* | F: GGT GAA CTT CAA GAT CCG CCA |
| *EGFP* | Genotyping *Fus(EGFPplin2)* | R: GAA CTC CAG CAG GAC CAT GT |
